# Engaging patients and family members to design and implement patient-centered kidney disease research

**DOI:** 10.1186/s40900-020-00237-y

**Published:** 2020-11-01

**Authors:** Teri Browne, Amy Swoboda, Patti L. Ephraim, Katina Lang-Lindsey, Jamie A. Green, Felicia Hill-Briggs, George L. Jackson, Suzanne Ruff, Lana Schmidt, Peter Woods, Patty Danielson, Shakur Bolden, Brian Bankes, Chelsie Hauer, Tara Strigo, L. Ebony Boulware

**Affiliations:** 1 College of Social Work, University of South Carolina, Columbia, SC USA; 2 Family member co-author, Edgewater, MD USA; 3 Department of Epidemiology, Johns Hopkins Bloomberg School of Public Health, Baltimore, MD USA; 4 Welch Center for Prevention, Epidemiology and Clinical Research, Baltimore, MD USA; 5 Present address: Alabama Agriculture & Mechanical University, Huntsville, AL USA; 6 Department of Social Work, Alabama A & M University, Huntsville, AL USA; 7 Department of Nephrology, Geisinger Commonwealth School of Medicine, Danville, PA USA; 8 Kidney Health Research Institute, Geisinger, Danville, PA USA; 9 Division of General Internal Medicine, Johns Hopkins University, Baltimore, MD USA; 10 Division of General Internal Medicine, Duke University School of Medicine, 200 Morris Street, 3rd floor, Durham, NC 27701 USA; 11 Family member co-author, Mooresville, NC USA; 12 Patient co-author, Liberty, IL USA; 13 Patient co-author, Hartsdale, NY USA; 14 Patient co-author, Portland, OR USA; 15 Patient co-author, Jacksonsville, FL USA; 16 Patient co-author, Bloomsburg, PA USA; 17 Center for Clinical Innovation, Institute for Advanced Application, Geisinger, Danville, PA USA

**Keywords:** Patient centered, PCORI, Kidney disease, Care transitions, Patient research partners, Family member research partners, Outcomes, Shared decision-making, Patient and public involvement

## Abstract

**Plain English summary:**

We need more research projects that partner and engage with patients and family members as team members. Doing this requires that patients and family members set research priorities and fully participate in research teams. Models for this patient and family member engagement as research partners can help increase patient centered outcomes research. In this article, we describe how we have successfully engaged patients with kidney disease and family members as Co-Investigators on a 5-year research project testing a health system intervention to improve kidney disease care.

**Abstract:**

**Background**

This article describes a method for successful engagement of patients and family members in all stages of a 5-year comparative effectiveness research trial to improve transitions of care for patients from chronic kidney disease to end-stage kidney disease.

**Methods**

This project utilized the Patient-Centered Outcomes Research Institute’s conceptual model for engagement with patients and family members. We conducted a qualitative analysis of grant planning meetings to determine patient and family member Co-Investigators’ priorities for research and to include these engagement efforts in the research design. Patient and family member Co-Investigators partnered in writing this paper.

**Results**

Patients and family members were successfully engaged in remote and in-person meetings to contribute actively to research planning and implementation stages. Three patient-centered themes emerged from our data related to engagement that informed our research plan: kidney disease treatment decision-making, care transitions from chronic to end-stage kidney disease, and patient-centered outcomes.

**Conclusions**

The model we have employed represents a new paradigm for kidney disease research in the United States, with patients and family members engaged as full research partners. As a result, the study tests an intervention that directly responds to their needs, and it prioritizes the collection of outcomes data most relevant to patient and family member Co-Investigators.

**Trial registration**

NCT02722382.

**Supplementary information:**

**Supplementary information** accompanies this paper at 10.1186/s40900-020-00237-y.

## Background

In the United States (U.S.), the inclusion of patients and family members as partners in the development and implementation of research studies has been significantly growing with the creation of the Patient-Centered Outcomes Research Institute (PCORI) in 2010. PCORI prioritizes research that examines topics and outcomes most important to patients and their family members and engages patients and family members as active collaborators on research teams [1]. This approach has the potential to increase the likelihood that health research is valued and useful to the people with the highest potential to be impacted by the study results and most impacted by the disease studied- patients and family members [2, 3]. The “patient-centered” approach to research in the U.S. is aligned with a long history of other international efforts to include patients and family members in research, such as the James Lind Initiative [4], patient and service user engagement approaches to research [5, 6], and public and patient involvement in research [7].

In the U.S., the shift towards patient-centered outcomes research (PCOR) is grounded in a broader movement in health care towards patient-centered care and shared decision-making, where patients are actively involved in making treatment decisions [8–12]. The U.S. Centers for Medicare and Medicaid Services (CMS) now prioritizes patient and family engagement in its policies and programs [13].

It is increasingly recognized that a patient-centered approach in kidney disease must occur to improve patient education and outcomes [14–20]. End-Stage Kidney Disease (ESKD) is a chronic illness that occurs when kidneys stop working and is usually caused by diabetes and hypertension [21]. ESKD is an international and American public health crisis. In the U.S., there were 746,557 people in 2019 who had ESKD, requiring dialysis or kidney transplantation [21]. In addition to the health and quality of life consequences of ESKD, this chronic disease also represents a very significant federal public health burden in the U.S, as more than $500.5 billion is spent each year for ESKD care [21].Until recently, there have been few examples of PCOR in kidney disease, and little national attention in the U.S. on kidney disease PCOR. The majority of kidney disease (73.18%) researchers in a 2017 National Kidney Foundation survey (*n* = 647) had not been involved in any research that directly engaged patients [22]. Encouragingly, PCOR is expanding in kidney disease with several studies using this approach to improve care [16, 23–33]. The international Standardised Outcomes in Nephrology (SONG) initiative has also resulted in numerous publications and guidelines for CKD patient-centered outcomes in research [34–37].

Kidney disease PCOR can help promote patient-centered care in kidney disease settings and can also help the kidney disease community meet the goals of the 2019 CMS Treatment Choices Model [38] and Advancing American Kidney Health initiative [39] that aim to improve patient outcomes. Recent research suggests that patients with kidney disease prioritize very different outcomes than health care professionals [25, 40–42], and including patients and family members as research partners may help address this discordance as well as improve kidney disease care quality [42]. People with kidney disease are also increasingly demanding that research and innovation must include patients and family members as research partners [43–45].

Successful PCOR requires that patients and family members engage as full partners in research projects. However, there can be challenges to realizing this engagement in kidney disease as it requires a new paradigm of patients and family members setting research priorities and actively participating in research teams, rather than just be “tokenistic” research team members [46] who are minimally involved. Despite the growing body of research related to kidney disease PCOR, there is a paucity of literature regarding the actual patient and family member engagement process in such studies. To our knowledge, no studies have described in detail the patient and family engagement approach of a kidney disease intervention study.

Efforts to share methods for engagement of patients and family members throughout the conduct of PCOR studies are needed to refine methods across the kidney disease community. To address this literature gap and help inform future kidney disease PCOR studies, this paper examines one overall approach to patient and family member research engagement. We describe our method for successful engagement of patients and family members as Co-Investigators in the development and implementation of a 5-year PCORI-funded study to evaluate the impact of a pragmatic health system intervention on transitions of kidney disease care for patients with advanced chronic kidney disease.

## Methods

Utilizing the approach that PCOR engagement must occur during every stage of research [47], our patient and family engagement efforts began in 2012 when we partnered with the American Association of Kidney Patients (AAKP) as a kidney disease organization stakeholder to submit a PCORI application. The AAKP is the oldest and largest fully independent kidney patient organization in the U.S. The AAKP identified eight patients with kidney disease and two family members from their membership who they knew were interested in working on a research project. The family members included the spouse of a patient with kidney disease and a living kidney donor who is a family member of multiple people with kidney disease. AAKP connected the author TB with these individuals, and TB communicated with them by phone and email to share the information about the PCORI funding mechanism, our interest in collaborating with them as partners on a grant application, and explained that we would have an initial conference call to discuss their ideas for research. TB discussed with them that their role on this project would be as full partners, and that we wanted to talk about their research question suggestions and how they wanted to be involved in the project we would develop together. All of these patients and family members were eager to be involved in this project, and agreed to work with us.

As our application developed, the team collectively decided that we needed to add a patient from the clinical implementation site we chose for our project- Geisinger Health System. Geisinger is () a health care system in the U.S. state of Pennsylvania and has a hospital as well as multiple regional clinics. The team thought that adding a patient from Geisinger would help ensure that we got feedback from someone who received care from the medical provider that is involved in the intervention study. Geisinger suggested a patient with kidney disease who was part of their advisory committee for this team, and author TB contacted the patient to explain the study and ask him if he was interested in working with us. This new Co-Investigator eagerly joined our meetings early in the planning process and was a full participant.

The eight patient and family members who joined the research team included five women (62.5%) and three men (37.5%), with an age range of 32–65 years (mean = 52.9, s.d = 10.3). The majority of them are white (75%). Compared to the demographics of the final study population [48], this group is younger (study mean age 71), more diverse (study mean ethnicity 97% white), and has similar gender distribution (study mean 59% women). However, at the time when we were assembling the study team, we did not have a study population in mind, therefore exact demographic similarities between the study team and research sample was not a priority. It was more important that the patient and family members had diverse geographic and kidney disease treatment experience.

These patients and family members lived across the U.S. (every one lives in a different state) and had a variety of kidney disease treatment experience (in-center hemodialysis, home hemodialysis, peritoneal dialysis, kidney transplant). Working with the AAKP to identify almost all of these patients and family members to be part of the research team, we collectively thought it was most important that the patient research team members had participated in different kidney disease treatments in order to understand and advise the project on the unique experiences of each treatment modality. We also thought it was critical to engage patients and family members from different geographical regions, as kidney disease practices can differ across the country.

### Conceptual framework for patient and family member engagement

This project utilized the PCORI conceptual model for engagement with patients and family members [49]. This conceptual model relies on seven PCOR principles: trust, honesty, co-learning, transparency, reciprocal relationships, partnership, and respect (see Table 1 for examples of how these concepts are operationalized). Utilization of this engagement model was essential because, before this project, we did not know any of the patients or family members. We came together as a team via a kidney disease stakeholder and had to create and build relationships in order to engage as a research team successfully.

**Table 1:** PCOR Engagement Principles and Examples

PCOR Engagement Principle	Team Actions
Trust	Worked with patient and family Co-Investigators over a long period of time, allowing everyone to get to know each other.
	Frequent communication and involvement in every aspect of our grant application.
Honesty	Approached our work with integrity.
	We were realistic about our capacity to accomplish study aims and possibility of grant rejection.
Co-learning	Learned from patient, family member suggestions for application details and incorporated these suggestions in final application.
Transparency	Shared every step of the application process and all materials.
Reciprocal relationships, Partnership	Fully depend on the patients and family members for the study idea, research aims, design and outcomes.
	PI personally asked each patient and family member to be a full Co-Investigator (Co-I) on our grant application.
Respect	We used terminology for the family members involved in our research team. We had a long conversation with this group about how to refer to the family members on our research team i.e. did they prefer to be called care partners, caregivers, or something else. Ultimately, they decided they wanted to be referred to as family members.

### Process for defining research study

As a team, we decided that we would conduct monthly internet/telephone conferences to identify a research topic, define specific aims, and develop a study design for a PCORI research application. We asked patients and family members what was most important to them that the collective team should study, and how we should go about conducting such research. Patients and family members shared their own and their family members’ challenges getting diagnosed with kidney disease and finding a treatment that was best for them. Collectively, we decided that we wanted to develop a study that would help us understand how we can improve the way patients transition from chronic kidney disease (CKD) to end-stage kidney disease (ESKD).

With the permission of attendees, we recorded and transcribed these meetings, and took minutes and notes. We conducted a qualitative analysis of these records to determine the patients’ and family members’ priorities for research and to include these engagement efforts in our PCORI application. We performed manual content analysis of the transcripts and documents by utilizing qualitative analysis techniques [50, 51], coding for content, and identifying relevant themes concerning the engagement process [52]. After this initial analysis, we created a list of the final themes through discussion with the patients and family members and reaching consensus. This manuscript abides by the Guidance for Reporting Involvement of Patients and the Public (GRIPP2) (see Additional file 1). Patient and family member Co-Investigators partnered in writing this paper.

## Results

**Fig. 1:**
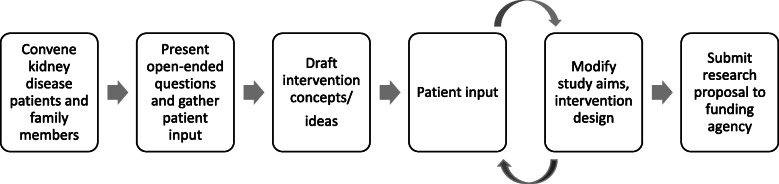
Process for Engaging Patients in Development of Research Proposal (Pre-Funding)

All of the patient and family member Co-Investigators participated in the research planning discussions and engagement process (see Fig. 1). After convening the Co-Investigators, we asked them open-ended questions about what research ideas were essential to them. We drafted research questions and a research design, and received more feedback from patients and family members that informed the final study design (see Table 2) [32]. Three patient-centered themes emerged from our data related to engagement that informed our research plan: decision-making, care transitions, and outcomes.

**Table 2:** Patient and Family Member Input that Guided Intervention Design

Domain	Questions and Ideas Presented by PI	Patient and Family Input	Final Study Design Changes Made
Overall Concept	What do patients need to help prepare them for decisions about which kidney disease treatment is best for them?	Patients should be asked what “quality of life” means for them, and need help getting the treatment that helps them achieve that quality.	Guided the creation of ‘Patient-Centered Kidney Transitions Care’ intervention. Includes: 1) tools to help providers focus on patients’ values and treatment preferences and 2) a Kidney Transitions Specialist who will provide patients with knowledge, skills and assistance.
		The whole point of “choice” is to continue your life as much as you want as possible. Patients need to be able to explore with their providers their goals and then receive information and education about the treatments that fit their lifestyle.	
	If patients are already on hemodialysis, what help is needed (if any?) to get a kidney transplant?	1. Many patients don’t know all their options for treatment. They are not given the choices.	Education and informed decision-making about kidney failure treatments are emphasized in the intervention.
		2. Patients often struggle with keeping an open mind about their treatment options. Hearing from other patients about their experiences on different treatments can help them to understand how treatment might work for them and their life.	Patients can be connected to peer support through a partnership with the National Kidney Foundation’s Peer Support Program.
		3. Need to create an educational video that covers the options for treatment and lets patients tell their story of being on treatment. Patients need to hear from other patients why they chose their treatment and what life is like on that treatment.	Incorporated decision support materials (video and book) including real patients talking about their experiences on the various kidney disease treatment modalities.
	How much information does a patient and family member need? What kind of support do people need when they get materials?	There are great materials out there and the intention is that someone will sit down and review them with patients, but the truth is that many of the staff doesn’t have the training or the time to do this.	Intervention adds Kidney Transitions Specialist to the health care team who can review materials with patients.
		There is variability in how much information a patient needs. Some patients are “hungry” for information but others can be turned off by too much information. Helpful to give patients a little bit of information at a time.	Kidney Transitions Specialist will ascertain patients’ current knowledge of kidney disease and offer individualized support.
	Could someone outside the treatment team serve as an advocate for the person making their own treatment choice?	Social workers have special training that helps them to talk with patients and explore with them their needs.	Led to creation of Kidney Transitions Specialist role.
Study participants	Target pre-ESKD patients who will likely need renal replacement therapy (RRT) within 1–2 years (eGFR < 18)	Too late in disease progression; want to have information earlier to slow progression to kidney failure.	Target pre-ESKD patients with eGFR < 30 or increased risk of disease progression determined by risk prediction model.
		What about targeting people with certain comorbid diseases who you think will progress to kidney failure?	
	Target patients who recently started dialysis	Too late in disease progression; once patients start dialysis it is hard to change treatment modalities.	No longer including patients who recently started dialysis.
Study Sites	Nephrology practices and dialysis facilities	Too late in disease progression to start at dialysis facilities; once patients start dialysis it is hard to change treatment modalities.	No longer intervening in dialysis facilities.
Intervention Component 1: Health Information Tools	Create a ‘registry’ of patients in the practice who, based on their eGFR or rate of kidney disease progression are likely to need to make a kidney disease treatment decision within 1–2 years, and give them information about treatment options.	Even “low risk” patients need some information so they are not blindsided if kidneys fail.	Stratify patients into low and high risk and tailor interventions accordingly.
	Care manager documents patients’ preference for kidney disease treatment before treatment initiation and physician signs off on preference (Advance directive of patient choice).	The patient would need a lot of information up front to be able to choose a treatment.	Patient receive needed treatment information from Kidney Transitions Specialist. Treatment decision included in medical record.
Intervention Component 2: New Team Member and Programs	Patient assigned to a care manager who helps the patient decide which kidney disease treatment is right for them.	Also want a buddy/peer to walk them through the process, like a college orientation. But also need care manager for technical info.	Kidney Transitions Specialist will act as their ‘champion’ and partner patients with peer support.
	All patients who get on the ‘registry’ are ‘prescribed’ the decision aids (books and videos)	Sometimes it is information “overload.” Need a human being there who could help them through the material and the process.	Kidney Transitions Specialist will meet 1-on-1 with patients for individual decision support.
	Nephrologist flagged to discuss kidney disease treatment options with patients on registry	Nephrologist only has 10 min with patients	Kidney Transitions Specialist will provide individualized, comprehensive support.
	Miscellaneous input	Patients go through grief cycle after kidney failure and have anxiety and depression, health providers don’t get that	Care manager supports patient and family psychosocially, helps them find support. Refer all patients to an initial mental health assessment and provide needed psychological support.
Outcomes	1. Patient Outcomes--Questionnaire: • Pre-ESRD choice of modality • Knowledge • Comfort • Family engagement • Decision-self-efficacy • Psychological: stress, depression, anxiety—global and specific to decision • Engagement in preparation activities (adherence to referrals) • Transplant evaluation and wait listing 2. Patient Outcomes—EMR: • Biomedical: BP control and anemia management after intervention • Circumstances of initiation and choice of initial therapy • Health care utilization 3. System Outcomes—EMR: • Care manager places order for preference in EMR • MD co-signs orders for preference KRT • Sustainability (RE-AIM)	1. Loss of Control – one of the hardest things for kidney patients to get their head around. “*To get control of the loss of control. How do you go on from here?”* Measure empowerment, purpose. 2. Acceptance – acceptance you will be on a machine. You have a choice to accept that you will be on that machine. Little things like needing to extend time on dialysis can be really devastating. 3. Grief – most of the medical team don’t get that patients are grieving a huge loss in their life. 4. Mental status 5. Quality of Life 6. Depression, Anxiety 7. Kidney understanding 8. Family member kidney understanding	Added to outcomes: • Patient Control, Decision-Making ○ Empowerment Score • Control (locus of control) and patient activation • Need for mental health support • Quality of Life

### Identified need to address decision-making

After extensive open discussions, the patients and family members came to a consensus that the most essential aspect of CKD care that needed improvement was decision-making. Patients and families felt there was not enough information and help for people diagnosed with kidney disease, and they were not prepared for kidney disease or choosing a treatment. Patients and family members thought that people with kidney disease needed education and information about kidney disease progression and related treatments as early as possible when diagnosed with CKD and that this information should be tailored to help people make kidney treatment decisions at different stages in the disease.

Every one of the patient and family member Co-Investigators had personal experience with this systemic problem. One of the family members summed this up, stating:

"I always think about cancer patients, no matter what stage they are at, they [doctors] sit down with them and the family, they talk about what stage they are at, what the options are, what may or may not happen, so that they can be fully prepared. But that doesn't happen with kidney patients and it should because it is a chronic illness and the person could end up on artificial life support or dead if they don't have the right options in place ahead of time. The same thing should be done for kidney patients."

A patient also stated,

"I still to this day cannot believe how ignorant and misinformed I was. This is why I volunteered for this study. I do not want another patient to go through what I went through."

### Identified need to support kidney disease care transitions

The patient and family member Co-Investigators believed that education and information were not enough to help people diagnosed with CKD, and they believed support was also needed at many stages of kidney disease care transitions. One patient stated, “as good as my doctor is, I still felt abandoned in the sea of information.” Together we decided that patients needed assistance and support to navigate treatment information because people who receive a CKD diagnosis feel overwhelmed with the amount of information to digest about kidney disease, kidney failure, and kidney disease treatments. The full team decided that patients need care transitions assistance as CKD progresses, and believed this assistance needed to be multifaceted. i.e., delivered by professionals, self-directed, class learning, and peer support. One of the family members stated in one of our meetings, “I wish such a strategy had been implemented when my husband was first diagnosed with kidney failure. Too many patients lack supportive services needed to carry the burden of their disease.”

### Identified patient-centered outcomes

Patient and family member research Co-Investigators identified patient-centered outcomes for the project that were most important to them: grief, anxiety, depression, acceptance, control, empowerment, and kidney disease knowledge. As people with kidney disease or family members of people with kidney disease, these Co-Investigators knew well the emotional impact of a CKD diagnosis. As one of the patients stated, he was “blind-sided” by his diagnosis of kidney failure. In team meetings, there were many conversations about how CKD can have a significantly negative impact on a patient and result in grief, anxiety, and depression. It was important to the team that the intervention addresses these common responses to a CKD diagnosis and that our project would help improve patient control, empowerment, and acceptance of CKD. It was also critically important to the team that the intervention helps improve knowledge about kidney disease and its treatment options.

### Intervention design

During research team meetings, we collaboratively designed an intervention to improve a health system (Geisinger Health) to respond to the critical themes of kidney disease decision-making and care transitions to improve patient-centered outcomes. The specific aims of the project directly reflect patients’ and family members’ suggestions and were refined by the entire team:

1. Establish a Patient-Centered Kidney Transitions Care infrastructure that (a) prioritizes kidney patients’ informed self-care and treatment decisions, and (b) supports patients through their transitions across kidney disease stages by providing education, psychosocial support, and biomedical preparation.
2. Study the effectiveness of the new Patient-Centered Kidney Transitions Care infrastructure to improve patients’ values-aligned kidney care, empowerment, and well-being.

This intervention would help patients with kidney disease make the best treatment decisions, have the best transitions across stages of kidney disease, and be designed to improve the outcomes most important to patients and family members.

### Engagement plan

Throughout the research application process, researchers were fully transparent and collaborative about all aspects of the process required to complete our submission. While compiling the PCORI application, it was essential to discuss the composition of the research team and the roles that the patients and family members would play on this team. As researchers, we believed that to fully embrace the PCOR principles, the patients and family members would be considered the true team experts and full partners on the research project. This would mean patients and family members would attend regular research team meetings and not serve only in an “advisory” role, but provide input on the study design and conduct, and be involved in study analyses and dissemination. Each patient and family partner accepted personal invitations via phone calls from the principal investigator to be a Co-Investigator in the study.

Finalizing our project engagement plan, we wrote into the application to PCORI that patient and family Co-Investigators would participate in weekly task committee and monthly full team project meetings using teleconference and internet conferencing technology. Also, they would participate in two in-person meetings at the end of Year 2 and Year 5 of the project. They would actively participate in all project workgroups, and patient and family Co-Investigator input would be solicited on all aspects of the study, including monitoring the conduct of the project, reviewing data collection procedures, data analysis, and interpretation of findings. Before submitting the proposal, patient and family Co-Investigators collectively decided that a rate of $35/h for their work on this project would be fair if the study were funded. We agreed that patient and family Co-Investigators would work approximately 102 h per year on the study (based on meeting frequency) and that their travel to in-person meetings would be funded by the study. If necessary, we also agreed to help patients schedule dialysis treatments for any study-related travel. The patient and family Co-Investigators helped write and edit several drafts of the PCORI application. The team was notified in 2015 that our project was funded for 5 years (https://www.pcori.org/research-results/2015/can-patient-centered-approach-preparing-patients-kidney-failure-improve).

### Ongoing engagement

**Fig. 2:**
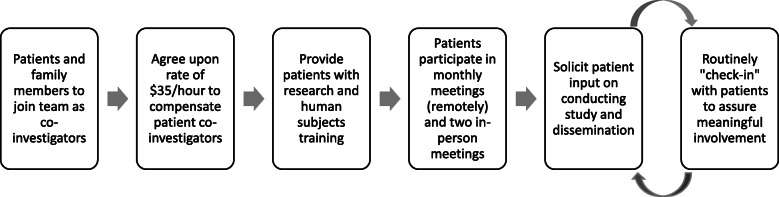
Process for Engaging Patients and Family Members on the Research Team

As we moved forward in the project (see Fig. 2) [32], patients and family members are compensated Co-Investigators, have been trained in the conduct of human subjects research, participate in team meetings and provide input on the study and intervention implementation. They also help disseminate information about the study. Co-Investigators write blogs for our website about their experiences having kidney disease as well as their experience in the research project (https://www.kidneypreparenow.org/blog). At the Co-Investigators’ request, we created and provided them project business cards and brochures to share at the kidney disease conferences and events they attend.

To encourage their engagement in the study, patient and family Co-Investigators all actively participated in weekly project workgroups during the initial year of the project during the intervention development phase (e.g., kidney transition workflows, patient materials for kidney education classes) and the development of processes to measure patient reported outcomes (e.g., telephone surveys). During the roll-out of the intervention, patients and family members continued to be engaged in the monitoring of the intervention by participating in monthly full team research meetings. The patient and family Co-Investigators also have monthly meetings to have regular “check-ins” to make sure they are meaningfully involved in and contributing fully to the study. One researcher Co-I is the main point person for this group and (along with project staff) routinely communicates with these Co-Investigators individually and as a group. Since project implementation, the patient and family Co-Investigators have been significantly engaged in every aspect of our research (see Table 3). This allows for our engagement process to continue to reflect the PCOR engagement principles.

**Table 3:** Patient and Family Co-I activities in each research phase

Research Phase	Patient and Family Member Co-Investigator Engagement Activities
Design: interventions/tools/comparators	Edited and finalized project logo and acronym.
	Suggested and designed an informational tool for the study that explains all of the different medical professionals patients may encounter when diagnosed with kidney disease.
	Intervention Work Group members chose and designed our interventions, all patient and family Co-Investigators provide feedback and suggestions in the design of our interventions.
Choice of outcomes and measures	Provided feedback and created project outcomes that were most important to them (loss of control, acceptance, grief, mental status, quality of life, depression, anxiety and understanding kidney disease)
	Helped design and refine our survey measure.
Participant recruitment	Reviewed recruitment materials and provided edits that were used in final recruitment materials (postcards, letters, fliers and telephone scripts)
	Made suggestions for increasing the study enrollees’ participation in the National Kidney Foundation peer support program.
	Edited new recruitment letters for education classes
Dissemination	Participated in our project website blog and social media to share information about our study: (http://www.kidneypreparenow.org), Facebook (https://www.facebook.com/KidneyPrepareNow/?fref=ts) and Twitter (https://twitter.com/Kidney_Prep_Now).
	Suggested and designed a project brochure that patient and family member Co-Investigators can take to kidney disease meetings and PCORI annual meeting.
	Presented as authors about the project at the American Society of Nephrology Kidney Week, American Association of Kidney Patients Annual Meeting, PCORI Annual Meeting and National Kidney Foundation Clinical Meeting. Co-author project journal article [16].

### Challenges

Some of the challenges in PCOR engagement have emerged as we have executed our study and project activities. First, some of the patient Co-Investigators receive in-center hemodialysis, and some work full-time, which limits their meeting availability on some days and times. We addressed this by having larger team meetings on different days to increase participation. We schedule Co-Investigator monthly meetings on a day and time that works for all, and adjust this schedule as needed throughout the year. Second, having a research team with members living all across the country also requires frequent and meaningful communication. To address this limitation, we have frequent email and phone conversations with the Co-Investigators and utilize video conferencing to share documents and materials that everyone can review at the same time. If materials are not available electronically, we mailed items to the Co-Investigators. We have many group conversations, but also, a researcher Co-Investigator has additional personal conversations with the patient and family Co-Investigators. At the start of the project, we also created a booklet of team member biographies that included everyone’s photo and personal details like hobbies/interests and why this project was important to everyone. This booklet was shared with all of the research team as a way to promote engagement across a team that had not met in person yet (these biographies are located on our project website https://www.kidneypreparenow.org/our-team.html).

A third challenge is that some patients and family members were initially reluctant to actively participate in meetings and deferred comments and opinions to the research professionals during team discussions. We addressed this challenge very early in meetings by telling the patients and family member Co-Investigators that they were the most important experts on this project, and we valued their expertise. We honored this commitment by following up on these sentiments and using their ideas in all aspects of the project and asking them to share their ideas in team meetings. We conduct group meetings using many open-ended questions and allowing the patients and family members to tell us what they wanted to research. As with any large group, some members may not participate as much as others, and we addressed this by explicitly asking for feedback from Co-Investigators who may not have shared an answer to a question or contributed to the meetings. Research teams that conduct patient-centered research have reported similar challenges [53], and our approaches for addressing these limitations may be helpful for other PCOR studies.

### Current activities

As our project ends its final year, the patient and family Co-Investigators continue to be fully engaged in all activities and review all study processes such as project implementation and study recruitment. They are also involved in all of the data analyses, interpretation and dissemination. Partnering with these patient and family Co-Investigators, we have submitted two additional PCORI applications to continue collaborative patient outcomes research. This project has created new opportunities for the Co-Investigators to be involved in research beyond this project. Several of the Co-Investigators are PCORI Ambassadors and have attended three PCORI national meetings. Two patient Co-Investigators now review grants for PCORI, and one patient Co-Investigator was invited to participate in the National Symposium on Renal Anemia Research sponsored by PCORI.

## Discussion

We provide an ongoing, successful model for engaging patients and family members in kidney disease PCOR. Building on the initial engagement approach used in our application process, we have actively included the patient and family Co-Investigators in every aspect of this project.

To date, we have experienced many positive outcomes with our engagement process. Actively engaging with patients and family members has increased the relevance of the study to address patients’ needs and the patient-centeredness of our intervention. Consistently, the patient and family member Co-Investigators on the study report high satisfaction with their participation in this research project.

The model we have employed to engage patients and family members as Co-Investigators in the PREPARE NOW study represents a new paradigm for kidney disease research, and could also be used for research on other chronic illnesses in the United State and beyond. This engagement has shaped the entire design of our research project from its inception. As a result of patient and family member engagement as true research partners, the study tests an intervention that directly responds to their needs, and it prioritizes the collection of outcomes most important to patient and family member Co-Investigators. It is essential to recognize that engagement “is a means not an end” [54] and that efforts must be made by researchers to incorporate the PCOR principles of trust, honesty, co-learning, transparency, reciprocal relationships, partnership, and respect from idea conceptualization to dissemination. Future research should also examine different aspects of kidney disease PCOR engagement in more depth. Further work to expand the model employed by PREPARE NOW to engage patients and family members could be beneficial in addressing patient-centered outcomes in kidney disease.

## Supplementary information

**Additional file 1.** GRIPP2 checklist.

## Data Availability

Not applicable.

## Abbreviations

AAKP: American Association of Kidney Patients
CKD: Chronic kidney disease
CMS: Centers for Medicare and Medicaid Services
ESKD: End-stage kidney disease
GRIPP2: Guidance for Reporting Involvement of Patients and the Public
PCOR: Patient-centered outcomes research
PCORI: Patient-Centered Outcomes Research Institute
U.S.: United States
