# Supplementary material for: Engaging patients and family members to design and implement patient-centered kidney disease research
Source: Res Involv Engagem. 2020 Nov 1;6:66. doi: 10.1186/s40900-020-00237-y (PMC7604920; doi:10.1186/s40900-020-00237-y)
Supplement: Supplementary file 1 — Additional file 1. GRIPP2 checklist. [file 40900_2020_237_MOESM1_ESM.docx]

GRIPP2 checklist

| **Section and topic** | **Item** | **Reported on page No** |
| --- | --- | --- |
| 1: Aim | Report the aim of the study | 7 |
| 2: Methods | Describe the methods used by which patients and the public were involved | 7-10 |
| 3: Results | Report the impacts and outcomes of PPI in the study | 11-18 |
| 4: Discussion and Conclusions | Summarise the main conclusions of the study | 19 |
| 5: Reflections/critical perspective | Include PPI, “patient and public involvement,” or alternative terms as keywords | 1 |
